# The Evolution, Oligomerization, Function, and Action Mechanism of α2-Macroglobulin

**DOI:** 10.3390/cells15040353

**Published:** 2026-02-15

**Authors:** Wenshuo Xie, Lili Gao, Hongkuan Deng, Dongwu Liu, Qiuxiang Pang

**Affiliations:** Anti-Aging & Regenerative Medicine Research Institution, School of Life Sciences and Medicine, Shandong University of Technology, Zibo 255049, China; xiewenshuo968@163.com (W.X.); gaoli96@sdut.edu.cn (L.G.); hkdeng@sdut.edu.cn (H.D.)

**Keywords:** α2-macroglobulin, evolutionary analysis, molecular structure, action mechanism, functions of α2-macroglobulin

## Abstract

α2-Macroglobulin (A2M), a large tetrameric glycoprotein with a molecular weight of approximately 720 kDa, is a key member of the α-macroglobulin superfamily. Its origin dates back 600–700 million years, positioning A2M as an evolutionary link within the α-macroglobulin family and complement components C3, C4, and C5. Structural predictions of A2M across different species reveal a remarkably high degree of conservation between invertebrates and vertebrates. A2M is abundantly present in the body fluids of both vertebrates and invertebrates, and its diverse biological functions are governed by five key functional domains within its molecular structure. The most well-established role of A2M is the entrapment and inhibition of proteases. Beyond that, it interacts with cytokines, growth factors, and membrane receptors, thereby playing a broad role in immune and inflammatory responses, hemostasis and coagulation, as well as in disease mechanisms and therapeutic processes. This review summarizes the origin and evolution of A2M, its molecular structure and functional domains, principal mechanisms of action, and research progress regarding its functions in both invertebrates and vertebrates. Our goal is to provide new insights and directions for further exploring the functional potential of A2M and its future applications in the treatment of clinical diseases.

## 1. Introduction

Innate immunity is an essential defense mechanism that enables the early detection of invading pathogens and initiates subsequent pro-inflammatory responses [1,2]. This system relies on a limited set of pattern recognition receptors (PRRs) expressed on host cells to identify and bind pathogen-associated molecular patterns (PAMPs), serving as the first line of defense against a wide range of infectious agents [3,4,5]. As a central component of innate immunity, the complement system is evolutionarily conserved across both invertebrates and vertebrates and plays a critical role in combating pathogenic microorganisms [3]. Key players in the complement pathway are thioester-containing proteins (TEPs). It is a superfamily that emerged early in animal evolution and has been identified in diverse organisms, including nematodes, insects, mollusks, fish, birds, and mammals. TEP members share a common evolutionary origin and exhibit high structural and functional conservation [2,6,7,8]. A defining feature of this family is the presence of a thioester (TE) motif, GCGE, which forms a unique intrachain β-cysteinyl-γ-glutamyl covalent bond, known as the thioester bond, between the side chains of cysteine and glutamate. This motif enables a range of conformation-sensitive binding interactions [2,9].

Based on structural and functional characteristics, TEPs are broadly classified into three categories: the α-macroglobulin superfamily, C3-like complement proteins, and macroglobulin complement-related (MCR) proteins [2,3,10]. Among these, α2-macroglobulin (A2M), a high-molecular-weight homotetrameric glycoprotein and key member of the α-macroglobulin superfamily, exhibits broad-spectrum protease inhibitory activity and functions as an acute-phase protein during immune responses [11,12]. To date, A2M has been identified in metazoans, including both vertebrates and invertebrates as well as in Gram-negative bacteria [13]. In humans, A2M participates in a wide array of biological processes. It not only regulates proteolysis but also enhances the proliferation and migration of immune cells and cancer cells [14,15]. It facilitates antigen uptake, processing, and presentation by antigen-presenting cells through membrane receptor interactions [16,17,18], acts as a carrier for cytokines and growth factors [19,20], and assists in clearing damaged extracellular proteins [21]. Additionally, A2M can function as a molecular chaperone under stress conditions, binding to misfolded pathogenic proteins and preventing their extracellular accumulation, particularly during innate immune activity [22,23]. Given its multifunctional roles across invertebrates and vertebrates, a comprehensive review and deeper investigation of A2M are warranted. This article systematically outlines the evolutionary origin of A2M, analyzes its key molecular structural features, elucidates the “protease trap” mechanism mediated by its bait region and thioester bond, and discusses the relationship between A2M function and human diseases. Ultimately, this review aims to provide new insights for developing A2M-targeted diagnostic and therapeutic strategies.

## 2. The Origin and Evolutionary Analysis of A2M

A2M emerged approximately 600–700 million years ago, predating the appearance of immunoglobulins [24]. Evolutionarily, the precursor of A2M was already present in invertebrates such as the horseshoe crab around 450 million years ago [25]. The hemolymph of the horseshoe crab (*Limulus polyphemus*) contains a thiol ester protein that participates in the hemolytic system and also exhibits protease inhibitory activity [26]. Its thiol ester is capable of binding both glycerol and methylamine, allowing the protein to serve as an evolutionary link between α-macroglobulin and complement components C3, C4, and C5 [27].

**Figure 1:**
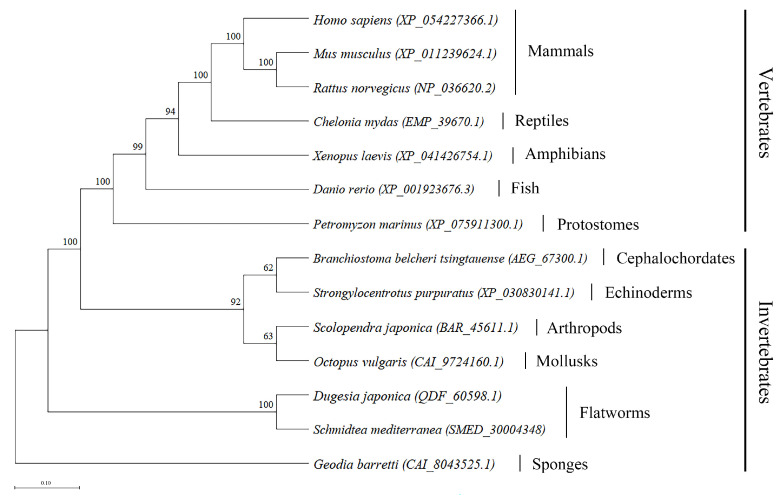
Phylogenetic tree of A2M constructed using MEGA11 software, with branches color-coded to represent different taxonomic groups.

**Figure 2:**
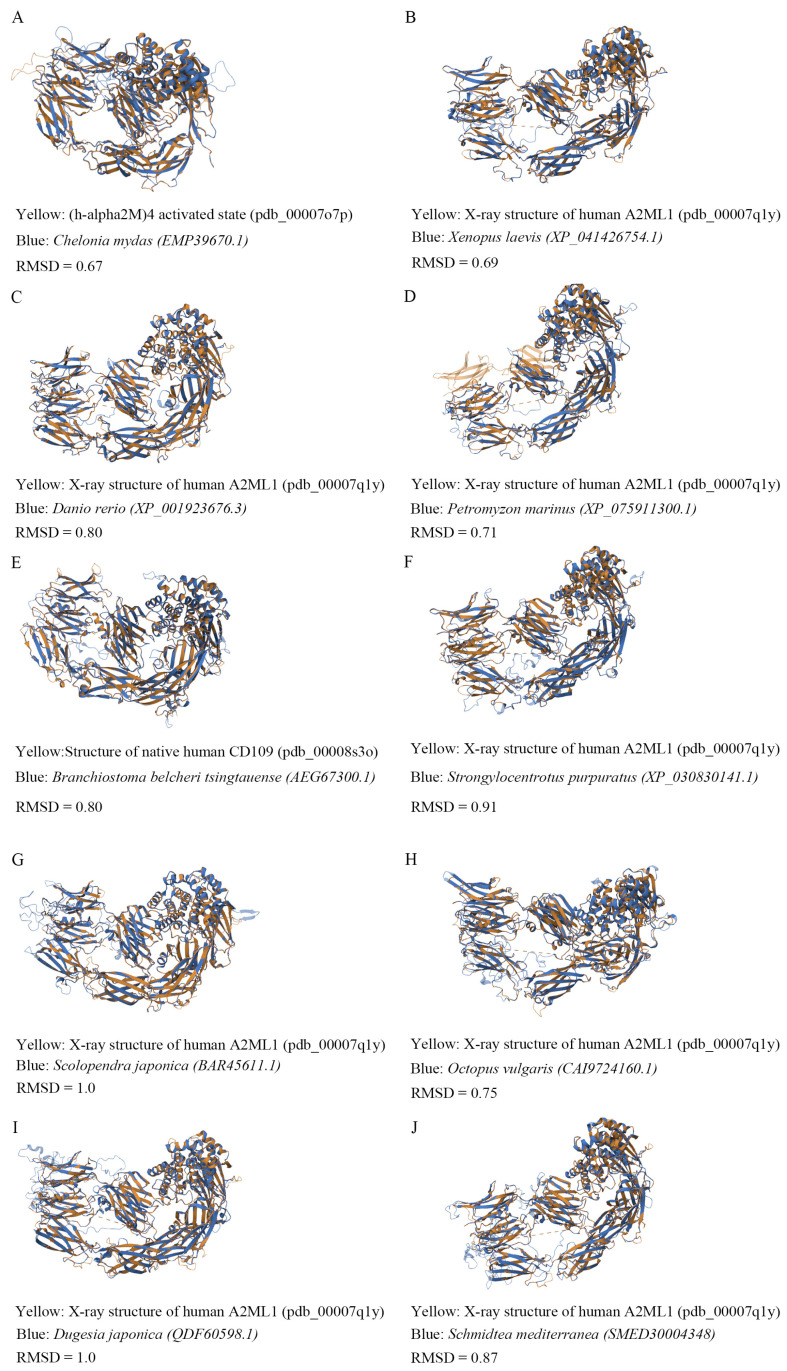
Comparison of the 3D structures of A2M from different animals. An RMSD below 3.0 indicates high structural similarity (**A**) Comparison of the 3D structures of (h-alpha2M) 4 activated state and *Chelonia mydas* (EMP39670.1). (**B**) Comparison of the 3D structures of the X-ray structure of human A2ML1 and *Xenopus laevis* (XP_041426754.1). (**C**) Comparison of the 3D structures of the X-ray structure of human A2ML1 and *Danio rerio* (XP_001923676.3). (**D**) Comparison of the 3D structures of the X-ray structure of human A2ML1 and *Petromyzon marinus* (XP_075911300.1). (**E**) Comparison of the 3D structures of the Structure of native human CD109 and *Branchiostoma belcheri tsingtauense* (AEG67300.1). (**F**) Comparison of the 3D structures of the X-ray structure of human A2ML1 and *Strongylocentrotus purpuratus* (XP_030830141.1). (**G**) Comparison of the 3D structures of the X-ray structure of human A2ML1 and *Scolopendra japonica* (BAR45611.1). (**H**) Comparison of the 3D structures of the X-ray structure of human A2ML1 and *Octopus vulgaris* (CAI9724160.1). (**I**) Comparison of the 3D structures of the X-ray structure of human A2ML1 and *Dugesia japonica* (QDF60598.1). (**J**) Comparison of the 3D structures of the X-ray structure of human A2ML1 and *Schmidtea mediterranea* (SMED30004348).

As a component of the innate immune system, A2M is highly conserved throughout evolution from invertebrates to vertebrates [28,29]. To investigate this conservation, we retrieved A2M protein sequences from various vertebrate and invertebrate phyla available on the NCBI website and constructed a phylogenetic tree using MEGA11 software with the maximum likelihood method (1000 bootstrap replicates). The resulting tree revealed high homology in A2M evolution across species, supporting its strong evolutionary conservation (Figure 1). We further predicted the tertiary structures of these A2M sequences using the Swiss Model, selecting the highest-scoring templates from the PDB database (Figure 2). The following templates were used: human Alpha-2-macroglobulin (AlphaFold DB: P01023.1.A) for *Homo sapiens* (XP_054227366.1); rat Alpha-2-macroglobulin (AlphaFold DB: P06238.1.A) for *Mus musculus* (XP_011239624.1) and *Rattus norvegicus* (NP_036620.2); human A2ML1 (X-ray structure: 7q1y.2.A) for *Xenopus laevis*, *Danio rerio*, *Petromyzon marinus*, *Strongylocentrotus purpuratus*, *Scolopendra japonica*, *Octopus vulgaris*, *Dugesia japonica*, and *Schmidtea mediterranea*; human activated A2M (7o7p.1.A) for *Chelonia mydas*; and human CD109 antigen (8s3o.1.A) for *Branchiostoma belcheri tsingtauense* (Table 1).

To assess structural similarity, we calculated root-mean-square deviation (RMSD) values between predicted models and their templates. An RMSD below 3.0 indicates high structural similarity [30,31]. Our analyses showed RMSD values below 1.0 for models of *Xenopus laevis*, *Danio rerio*, *Petromyzon marinus*, *Strongylocentrotus purpuratus*, *Octopus vulgaris*, and *Schmidtea mediterranea* when compared to the human A2ML1 structure (Figure 2B–D,F,H,J). Values of 1.0 were obtained for *Scolopendra japonica* and *Dugesia japonica* (Figure 2G,I), still reflecting high structural conservation. Additionally, the model of *Chelonia mydas* showed an RMSD of 0.67 against the activated human A2M structure (Figure 2A), and *Branchiostoma belcheri tsingtauense* had an RMSD of 0.8 against human CD109 (Figure 2E). In summary, the low RMSD values across a wide range of species confirm that A2M maintains a high degree of structural similarity throughout evolution, underscoring its strong structural conservation.

## 3. Oligomerization of A2M

In body fluids, α-macroglobulins consist of identical subunits and exist in monomeric (e.g., α1-inhibitor 3 in rats and hamsters, Murinoglobulin in mice), dimeric (e.g., pregnancy zone protein and invertebrate A2M), and tetrameric forms (e.g., A2M in most circulating body fluids and egg white) [27,32]. The predominant form of A2M is a large tetrameric glycoprotein with a molecular weight of approximately 720 kDa [33]. It is composed of four identical subunits, each about 180 kDa [34,35]. In humans, A2M is an extracellular secretory protein mainly synthesized in the liver and released into the bloodstream [36,37]. Although predominantly present in plasma, it is also found at low levels in cerebrospinal fluid and saliva [35,38,39,40]. The cage-like structure of A2M is assembled from four subunits, each comprising 1451 amino acids and forming a glycosylated multi-domain polypeptide chain [33]. Assembly begins with the formation of two covalently linked heterodimers via symmetrical interchain disulfide bonds. These dimers then associate non-covalently in an antiparallel, head-to-tail orientation to form the mature tetrameric quaternary structure [21,33]. Each A2M tetramer can bind two smaller protease molecules or one larger protease molecule [41]. Every A2M subunit contains 11 intrachain disulfide bonds. Under limited reduction or pH/salt-induced conditions, the tetramer can reversibly dissociate into dimers while largely retaining its overall conformation and leaving the inactive thioester bond unreacted (the functional role of the thioester bond will be discussed later). These dimeric and even monomeric forms remain active against peptidases, exhibiting properties similar to those reported for natural monomeric and dimeric homologs [27,42,43,44]. However, upon interaction with peptidases or under chemical induction, monomers and dimers tend to reassociate into more stable dimers or tetramers, suggesting that the induced tetrameric form is more resistant to dissociation and exhibits tighter binding [45]. Monomeric A2M can also form stable extended dimers following induction by specific peptidases [46]. The tetrameric A2M, a common inhibitor of active endopeptidases across all catalytic classes [47], primarily relies on the following five functional domains for its inhibitory mechanism.

## 4. Functional Domains of A2M

### 4.1. Bait Region Domain (BRD) and Bait Region

**Figure 3:**
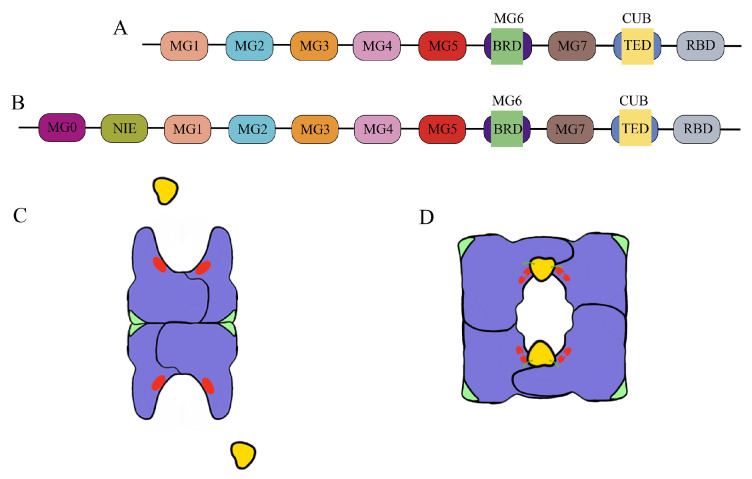
Schematic representation of the molecular structure of A2M and its tetrameric conformation. (**A**) Domain architecture of human A2M, depicting the MG1–MG7 domains, CUB domain, RBD, and the insertion positions of the BRD and TED. (**B**) Domain organization of a bacterial A2M homolog, highlighting the additional MG0 and NIE domains unique to bacteria, alongside the conserved MG1–MG7, CUB, RBD, and insertion sites for BRD and TED. (**C**) Native A2M in its protease-free state (proteases represented as yellow shapes), composed of four identical subunits. Each monomer contains an exposed bait region (red line) and a buried RBD (green structure). (**D**) Transformed A2M after protease cleavage of the bait region (indicated by a broken red line). Cleavage triggers a conformational change that entraps the protease and exposes the thioester bond, enabling covalent linkage to the protease (green curve). Concurrently, the RBD becomes surface-accessible, allowing the transformed A2M complex to bind cell surface receptors.

A2M comprises seven macroglobulin-like domains (MG1–MG7; Figure 3A), each adopting a seven-stranded antiparallel β-sheet sandwich fold composed of a three-stranded and a four-stranded β-sheet. Together, these domains constitute the central structural scaffold, into which other structural elements are inserted. The length of individual MG domains varies from approximately 70 to 128 residues. Bacterial homologs additionally contain two N-terminal domains, MG0 and the N-terminal Induced Domain of *Escherichia coli* α2-Macroglobulin (NIE; Figure 3B) [46,48,49]. The bait region domain (BRD) is inserted within MG6 and spans approximately 66–126 residues, adopting an irregular fold stabilized by three α-helices and interactions with neighboring MG domains and the C-terminal receptor-binding domain (RBD). The BRD also engages structurally with MG1, MG2, MG3, and MG5 [50]. It exhibits an extended conformation that renders it highly susceptible to cleavage by endogenous or exogenous endopeptidases [13,27,51]. Its principal role is to maintain the bait region in an extended, solvent-exposed conformation, facilitating access and cleavage by target proteases [33,46]. The bait region itself is a 25-amino-acid segment located within the BRD and exposed at the center of each A2M subunit. Cleavage of this region exposes internal thioester bonds, triggering a large-scale conformational change in A2M that enables protease entrapment. Thus, the BRD functions as a molecular switch that initiates A2M activation. Although amino acid substitutions in the BRD can preserve overall functionality, they may alter specificity toward non-target proteases, such as the Tobacco etch virus protease [52]. Notably, the eukaryotic BRD is substantially larger than its bacterial counterpart, suggesting evolutionary diversification that may enhance selective recognition of pathogenic endopeptidases while avoiding interference with host proteases involved in normal physiological processes.

### 4.2. Thioester Domain (TED)

Downstream of MG7 lies a C1r/C1s, Uegf, and Bmp1 (CUB) domain, comprising 116 amino acid residues folded into an all-β structure [33]. Inserted between the β3 and β4 strands of the CUB domain is the thioester-containing domain (TED), positioned beneath the CUB domain. The TED is composed of 315 residues and adopts an α-α loop surface helix architecture. It consists of six concentric α-hairpins arranged into a hexameric α-propeller around a central axis, forming a thick disc with two parallel faces (the entrance and exit faces) defined by the N- and C-termini of the intra-loop helices, respectively [13,33]. The TED is structurally adjacent to MG1 and MG2, contributing to the overall compact conformation of A2M monomers.

In *Escherichia coli* α_2_-macroglobulin (ECAM), the TED segment contains the sequence C-L-E-Q [48], which forms an internal β-cysteinyl-γ-glutamyl thioester bond between the side chains of cysteine and glutamic acid [53]. This bond is targeted by lysine residues on the surface of proteases, leading to covalent cross-linking. In contrast, the TED sequence in humans and other mammals is C-G-E-Q. In its native state, the TED in mammalian A2M contains a buried thioester bond, also referred to as a reactive β-cysteinyl-γ-glutamyl thioester, formed between cysteine and glutamic acid side chains [50,54]. This thioester bond is labile and can be cleaved by heat, primary amines, reducing agents, and other small nucleophiles, including water [50,53]. While methylamine is commonly used to activate A2M experimentally, proteases perform this role in vivo. Cleavage of the thioester bond generates reactive glutamyl and cysteinyl residues in each A2M subunit. The glutamyl group rapidly reacts with water, leading to swift decay of its activity, though it can form a covalent ε-lysyl-γ-glutamyl cross-link with lysine residues on target proteases. Meanwhile, the cysteinyl group remains active and is capable of binding cytokines or toxins such as the ricin A chain [54,55,56,57]. Notably, some proteases effectively inhibited by tetrameric A2M do not form covalent bonds with it. This may occur because these proteases (e.g., human neutrophil elastase) lack accessible surface lysine residues [58], or because the exposed thioester bond is rapidly hydrolyzed by solvent [59]. The electrophoretic mobility of A2M varies depending on the status of the thioester bond, leading to nomenclature based on these forms: when the thioester is intact, A2M migrates more slowly and is referred to as “native,” “slow,” or “active” (Figure 3C); after cleavage, it migrates faster and is termed “transformed,” “fast,” or “inactivated” (Figure 3D) [60].

### 4.3. Receptor-Binding Domain (RBD)

Following the formation of the TED, the polypeptide chain reconnects with the CUB domain and extends toward the C-terminus to form the receptor-binding domain (RBD) [33]. The RBD is situated adjacent to the CUB domain, near MG3, and interacts with both the TED and CUB. Structurally, the RBD adopts a β-hairpin fold (a variant of the MG domain) that incorporates an additional β-α-β motif, also referred to as the MG8 domain [33,42]. This domain remains stable across a broad pH range (2.5–9.0) and exhibits high resistance to proteolysis. Within the RBD lies a receptor-binding site that remains concealed in the native, uninduced state of A2M, preventing interaction with cellular receptors. Upon activation and the associated conformational change in A2M, the RBD becomes exposed on the molecular surface, revealing the binding site. This mechanism ensures that only A2M–protease complexes are targeted for clearance, while native A2M remains unaffected. Once exposed, the RBD is recognized by the ligand-binding domain of the low-density lipoprotein receptor-related protein (LRP). LRP is a 600 kDa endocytic membrane glycoprotein and a key member of the low-density lipoprotein receptor superfamily [34,61,62,63]. Recognition by LRP triggers receptor-mediated endocytosis of the A2M–protease complexes, leading to their internalization and subsequent degradation within lysosomes, thereby facilitating rapid clearance from the system [35,50,64,65,66].

### 4.4. The Transglutaminase Reactive Site

A2M serves as a major transglutaminase (TGase) substrate in human serum [32]. The TGase reaction site in A2M is situated near the bait region, approximately 20 amino acids upstream of the primary protease cleavage site, and remains exposed even in the native, uninduced conformation. This accessibility allows TGase to catalyze the formation of irreversible covalent bonds between A2M and primary amines or peptide segments [27].

### 4.5. The Zinc-Binding Site

A2M is a metalloprotein and the principal zinc-binding protein in plasma [67]. While zinc is dispensable for its protease-binding (antiprotease) activity, it is essential for the interaction with interleukin-1β (IL-1β) [27,32].

## 5. The Mechanism of Action of A2M

A2M is a major and ubiquitous protease inhibitor capable of inactivating a wide spectrum of proteases [34]. Its inhibitory mechanism differs across species: in bacteria such as *Escherichia coli*, A2M inactivates proteases through covalent binding and steric hindrance, whereas in humans, other mammals, birds, amphibians, and invertebrates like snails, A2M does not function like conventional protease inhibitors. Rather than blocking the active site of proteases directly, A2M employs its tetrameric structure to encapsulate active proteases within a cage-like conformation, effectively “physically isolating” them. Although the encapsulated protease remains catalytically active, its access to macromolecular substrates is prevented. Notably, small-molecule substrates or inhibitors can still enter the cage and interact with the trapped protease [33,50]. Monomeric and tetrameric forms of A2M utilize two distinct mechanisms for protease entrapment, referred to as the “Snap-trap” mechanism and the “Venus-flytrap” mechanism, respectively [50].

**Figure 4:**
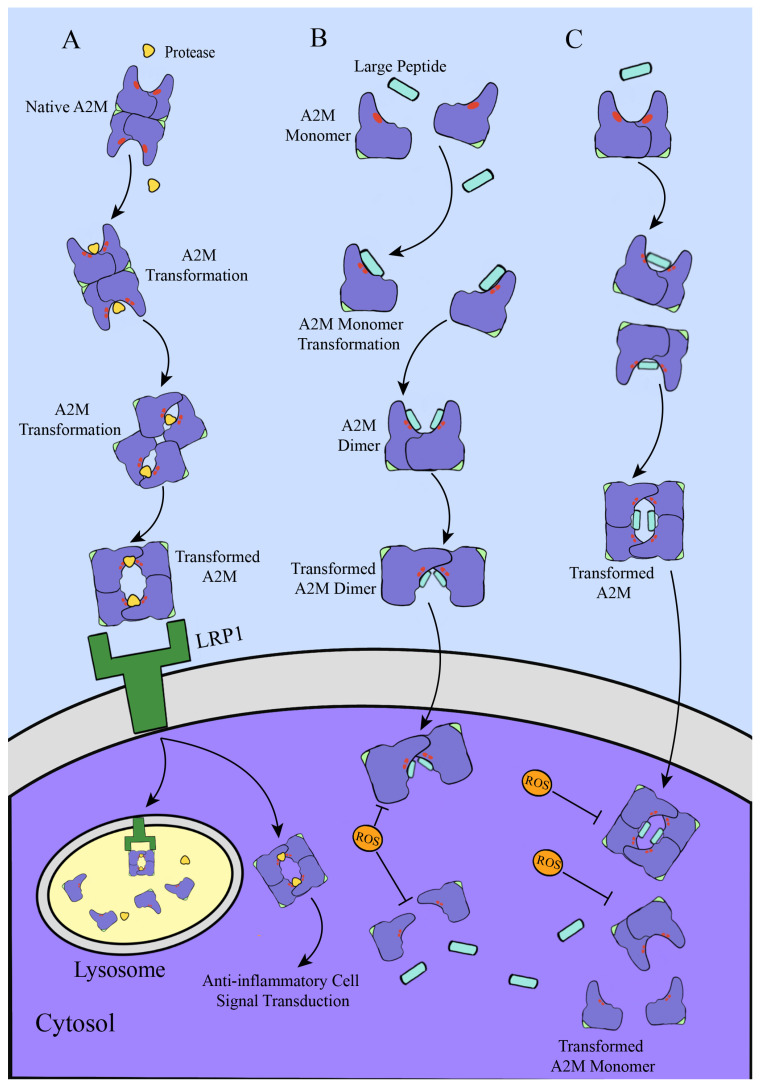
(**A**) Primary mechanism of protease capture by tetrameric A2M. Upon binding to a target protease (yellow shapes), native tetrameric A2M undergoes a conformational change. Cleavage of the bait region (red line) by the protease triggers exposure of the thioester bond, which forms a covalent linkage with a lysine residue on the protease surface. This is followed by further structural rearrangement of A2M, leading to the exposure of the receptor-binding domain (RBD) (green structure) on the tetramer surface. The transformed A2M–protease complex is then recognized by the LRP1 receptor, internalized via receptor-mediated endocytosis, and targeted to lysosomes for degradation. (**B**) Peptide capture mechanism of monomeric A2M. Monomeric A2M exhibits a more open structure, allowing peptides (light blue rectangle) to freely access and bind to its thioester bond via lysine residues. Following peptide binding, the A2M monomer can reassemble into a dimer for cellular entry. Intracellular reactive oxygen species (ROS) can inhibit the function of this dimer or promote its dissociation into monomers. (**C**) Peptide capture mechanism of dimeric A2M. After binding to a peptide, dimeric A2M can further assemble into a stable tetramer for cellular internalization. Certain intracellular reactive oxygen species (ROS) may inhibit the function of this tetramer or induce its dissociation into dimers or monomers. Arrows indicate mechanistic processes, while T-shaped arrows represent inhibitory effects.

The tetrameric form of A2M captures proteases via the “Venus-flytrap” mechanism [33], which involves a sequence of “binding–inhibition–tagging–endocytosis–degradation.” In this process, proteases enter the open structure of native A2M through a central pore in the tetramer, which is structurally permissive to protease internalization [33,68]. The protease then binds and cleaves the flexible and accessible bait region domain (BRD) located in the middle of the polypeptide chain [37]. The bait region in mammalian A2M contains multiple potential cleavage sites; proteases that cannot cleave this region are either rare or too large to be internalized [52]. Following cleavage of the multi-target bait region, the protease inhibition process is initiated. This triggers a conformational change in A2M, leading to an irreversible interaction with the protease and ultimately resulting in its entrapment [11]. Here, BRD is cleaved by the target protease, leading to the burial of the TED. This exposes a highly reactive β-cysteinyl-γ-glutamyl thioester bond, which then reacts with a lysine residue on the surface of the target protease. Upon cleavage of the thioester bond, free glutamyl and cysteinyl residues are released, and a covalent linkage forms between the glutamyl residue and the lysine of the protease. As a result, the A2M tetramer becomes covalently cross-linked to the target protease [13,27,69]. This breakdown of the thioester bond induces further conformational changes in A2M, leading to the exposure of the receptor-binding domain (RBD) on the surface of the tetramer. The transformed A2M–protease complex is then specifically recognized by the low-density lipoprotein receptor-related protein (LRP) on the surface of various cell types, including fibroblasts, macrophages, vascular smooth muscle cells, hepatocytes, and neurons. The complex is internalized via receptor-mediated endocytosis and degraded in lysosomes, enabling rapid clearance from circulation within minutes of complex formation (Figure 4A). This process also exerts cytoprotective effects and participates in anti-inflammatory signaling [33,70,71]. Notably, native A2M is not recognized by LRP because its RBD remains buried. However, tetrameric A2M can undergo a similar conformational rearrangement when induced by small primary amines such as methylamine, which also cleave the thioester bond. This reaction proceeds more slowly than protease-induced activation and yields a free cysteine thiol and an N-substituted glutamine residue. In human tetrameric A2M, even if the bait region remains intact, cleavage of the buried thioester bond by small nucleophiles like methylamine renders the molecule unable to react with or capture endopeptidases, thereby abolishing its inhibitory activity [50,72].

Currently, the term “Snap-trap” mechanism has two interpretations. One refers to the protease inhibition mechanism of bacterial A2M (e.g., in *E. coli*), which relies on covalent binding and steric hindrance and is effective mainly against very large substrates [50]. The other describes the mechanism by which monomeric A2M captures proteases [33,46]. In monomeric and dimeric forms, the bait region protrudes outward and is fully exposed, allowing proteases to access it freely [46,48]. This structural openness enables a broader inhibition spectrum against proteases of varying sizes. Whether a protease is inhibited in this context depends on the presence of a surface lysine residue available for cross-linking via the thioester bond. The more accessible structure of monomeric and dimeric A2M also facilitates interaction with larger peptide substrates compared to the tetrameric form [46,73]. However, when monomeric or dimeric A2M reacts with proteases or is chemically induced, it tends to reassemble into more stable dimers or tetramers, suggesting that the induced tetrameric form has stronger binding and is less prone to dissociation. On the other hand, reactive oxygen species (ROS) released by neutrophils may compromise the structural integrity of A2M, causing tetramers to dissociate into dimers and leading to a significant reduction or complete loss of protease inhibitory activity [74,75] (Figure 4B,C). Whether monomeric or dimeric A2M retains protease inhibitory activity under physiological conditions remains to be fully validated through further experimental investigation.

## 6. Functions of A2M

### 6.1. Functions of A2M in Invertebrates

A2M is found not only in vertebrate body fluids but has also been cloned and identified in numerous invertebrates. The first invertebrate A2M to be reported was from the arthropod horseshoe crab. Since then, A2M has been identified in various other arthropods, including the Atlantic shrimp [76], white shrimp [77], crayfish [78], blue crab [79], Chinese mitten crab [26], and barnacle [80]. Beyond arthropods, A2M has also been characterized in cephalochordates [81], cnidarians [82], hemichordates [83], and mollusks [84]. As invertebrates rely primarily on innate immunity, A2M expressed in their hemocytes has been shown to serve not only as a broad-spectrum protease inhibitor but also as an important modulator of innate immune responses. For example, in pearl oysters, A2M can bind to Vibrio alginolyticus and significantly inhibit its growth. It also enhances hemocyte phagocytosis upon pathogen binding, thereby improving host resistance [85]. In the swimming crab (Portunus trituberculatus), A2M activates the phenoloxidase system, a key immune component that generates antimicrobial quinones, promoting phenolic substrate oxidation and strengthening hemocyte phagocytosis. A2M also upregulates antimicrobial peptide expression, further enhancing antibacterial capacity [86]. Similarly, in shrimp, the interaction between hemocyanin and A2M fine-tunes phenoloxidase activity, enabling effective immune activation while preventing excessive or damaging immune reactions [87]. These findings clearly demonstrate that A2M contributes directly or indirectly to innate immune defense in invertebrates.

To further elucidate the functional mechanisms of A2M in invertebrates, researchers have identified and studied several of its interacting proteins. In hard ticks, pathogen-derived metalloproteases influence the phagocytic clearance of Chryseobacterium. Knockdown of A2M significantly reduces this phagocytic efficiency, indicating that A2M interacts with pathogen proteases and plays a key role in bacterial clearance [28]. In shrimp, although hemocyanin alone activates phenoloxidase and A2M alone suppresses it, their combined interaction allows precise regulation of the immune response, suggesting that antibacterial activity depends on synergistic control by both proteins [87]. Recent work in echinoderms has revealed that A2M not only binds fungi and bacteria, exerting broad-spectrum antimicrobial activity, but also interacts with glucose-regulated protein 78 (GRP78), enhancing hemocyte phagocytosis and modulating endoplasmic reticulum stress to amplify immune responses [88]. The conserved immune-regulatory functions of A2M across species highlight its evolutionary significance in host defense. Nevertheless, research on invertebrate A2M remains at an early stage, and its full role and mechanisms are not yet fully understood. Further investigation into the functions and pathways of A2M in invertebrate immunity will be essential to clarify its key position in innate immune systems and may provide insights relevant to human immune enhancement and disease prevention.

### 6.2. Functions of A2M in Vertebrates

#### 6.2.1. A2M as a Protease Inhibitor

A2M is a broad-spectrum protease inhibitor whose primary function is the clearance of exogenous and excess endogenous proteases from tissues. It is capable of inhibiting nearly all protease classes, including serine, thiol, carboxyl, and metalloproteases [27]. As noted earlier, A2M specifically binds to catalytically active proteases. Unlike conventional protease inhibitors, it does not block the protease active site but instead entraps the protease within its molecular structure, sterically hindering access to macromolecular substrates while still permitting small molecules to enter and react with the protease. Consequently, A2M-bound proteases retain catalytic activity toward small substrates. Through these protease interactions, A2M participates in human immune and inflammatory processes and influences a wide range of physiological and pathological pathways.

A key role of A2M is the rapid inhibition of excess proteases released during tissue injury. For instance, it effectively inhibits major proteases released by neutrophils—including neutrophil elastase (NE), proteinase 3, cathepsin G (CatG), and matrix metalloproteinase-9 [66,89]. During phagocytosis and neutrophil turnover, these proteases are released into the extracellular space, where their activity must be tightly regulated to prevent degradation of connective tissue components such as elastin, collagen, and proteoglycans [90]. This regulatory function is particularly relevant in inflammatory diseases, where A2M helps prevent structural damage by neutralizing proteases from activated leukocytes [91]. A2M also inhibits proteases secreted by invading microorganisms, thereby limiting infection and parasitic growth across species [92]. Furthermore, A2M enhances macrophage phagocytosis and antimicrobial activity, facilitates microbial antigen presentation to macrophages, and promotes immune activation [66]. In arthritis, A2M levels correlate positively with the degree of inflammation. Studies have identified A2M–protease complexes (with NE and CatG) in joint cavities, and intra-articular injection of A2M in a collagen-induced arthritis mouse model significantly reduced inflammation [93].

A2M also shows promise as a therapeutic target and diagnostic marker. In Parkinson’s disease models, elevated A2M levels enhance inhibition of NE activity, influencing neuroinflammation and oxidative stress [94]. A2M also exhibits anticoagulant activity through inhibition of thrombin and coagulation factor Xa, suggesting a potential role in thrombosis treatment [34]. Salivary A2M correlates with blood glucose and glycated hemoglobin levels, indicating its utility as a potential biomarker for glycemic control [40]. Recent studies further propose serum A2M as a biomarker for type 2 diabetes in obese individuals [95]. However, elevated A2M levels may also impair physiological function. High A2M is considered a risk factor for cardiovascular disease, particularly coronary heart disease [96]. Together, these findings underscore the significance of A2M in the diagnosis, treatment, and risk assessment of numerous diseases.

#### 6.2.2. A2M Binds to Cytokines and Growth Factors

Cytokines serve as essential signaling molecules that regulate a wide range of physiological and pathological processes. A2M can bind to numerous cytokines, including basic fibroblast growth factor (bFGF) [97], platelet-derived growth factor (PDGF) [98], nerve growth factor (NGF) [99], IL-1β [54], and interleukin-6 (IL-6) [100], and modulate their biological functions. The effects of A2M binding vary among cytokines, and different cytokine–A2M interactions may involve different conformational forms of A2M (Table 2) [27]. While binding to native A2M can help stabilize certain cytokines in circulation, it may also facilitate their recognition by A2M receptors and subsequent clearance. Thus, cytokine activity is invariably influenced by complex formation with A2M (Table 3). For example, A2M binding protects IL-6 from degradation, enabling its sustained participation in host defense mechanisms such as immune and acute-phase responses, as well as hematopoiesis [100]. In sickle cell disease, the interaction between IL-6 and A2M has been shown to help modulate inflammatory homeostasis [101], supporting the notion that A2M helps maintain IL-6 activity under pathological conditions. Neurotrophins, on the other hand, preferentially bind to the transformed conformation of A2M [102]. A recent study by Geogres et al. demonstrated that both native and transformed A2M can form complexes with brain-derived neurotrophic factor (BDNF), inhibiting tropomyosin receptor kinase B activation and blocking BDNF-induced platelet aggregation [103]. This finding not only offers new insights into hemostasis and cardiovascular therapy but also highlights the functional relevance of A2M–neurotrophin interactions. Growing evidence indicates that A2M helps regulate key physiological processes through its association with cytokines and growth factors. In the cornea, A2M prevents TGF-β–mediated cataract formation [104], whereas in bone, it promotes osteoblast differentiation via TGF-β signaling [105]. Cardiac-specific A2M can also bind growth factors and corresponding receptors to support cardiomyocyte growth [106]. Recently, a novel engineered A2M derivative was developed by fusing a TNF-α–targeting peptide with the A2M receptor-binding domain. This simplified A2M protein acts as a TNF-α trap, promoting its internalization and lysosomal degradation. It has shown therapeutic potential in osteoarthritis by reducing cartilage degradation and inflammation, and in myocardial infarction by preserving cardiac function and limiting injury [107]. Beyond cytokines, A2M also interacts with hormones and influences endocrine-related pathways. It binds glucocorticoids and has been implicated in oxidative stress-induced femoral head necrosis [108], and modulates osteoblast differentiation via adrenocorticotropic hormone [105]. Furthermore, environmental estrogens can regulate A2M expression through estrogen receptor signaling, revealing a potential mechanism for mitigating estrogen-related oxidative stress [109]. Collectively, these studies demonstrate that A2M interacts with a variety of cytokines, growth factors, and hormones to help maintain homeostasis. However, the specific conformational requirements for these interactions remain unclear and warrant further investigation.

#### 6.2.3. Interaction of A2M with Membrane Receptors

**Figure 5:**
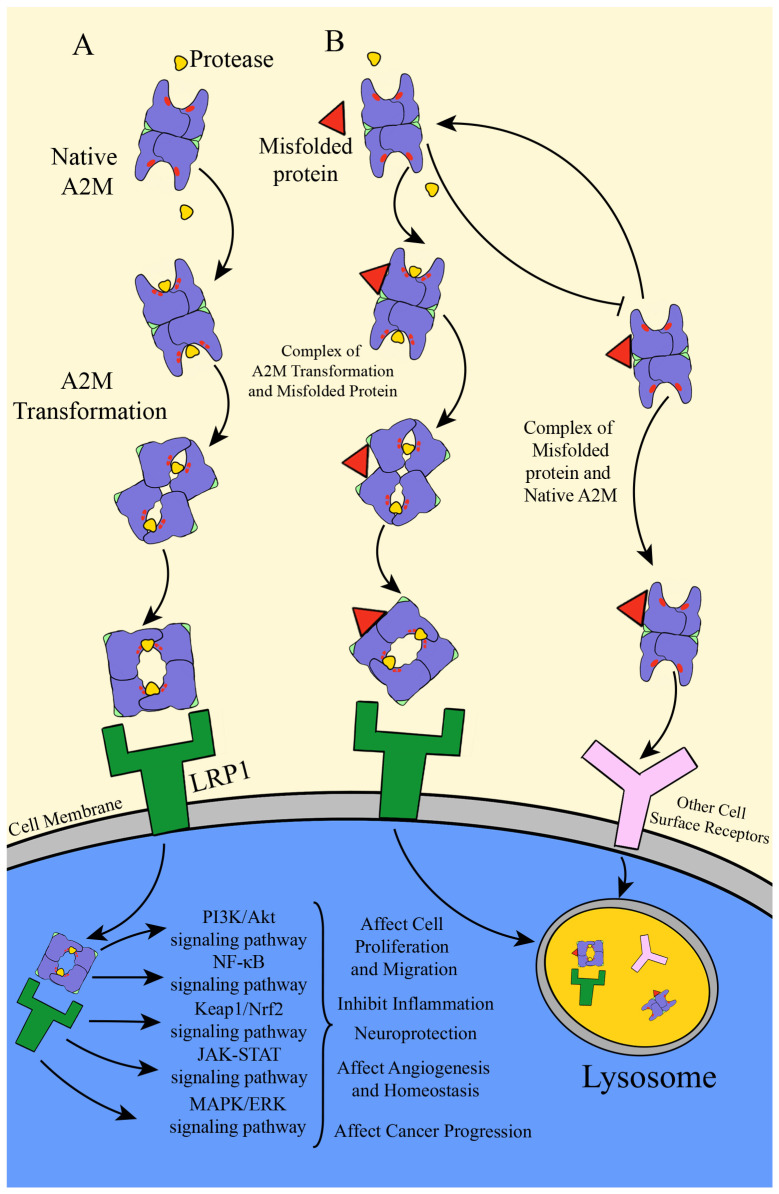
Mechanisms underlying the dual functions of A2M as a receptor ligand and molecular chaperone. (**A**) As a protease inhibitor, A2M binds to proteases (yellow shapes) and undergoes a conformational change, followed by interaction with the LRP1 receptor for cellular entry, where it cooperates with various signaling pathways to regulate cellular functions. (**B**) As a molecular chaperone, A2M first binds to misfolded proteins (red triangle) to form a complex, which does not activate the protease inhibitory activity of A2M. This complex can either bind to its corresponding receptor for cellular entry to exert biological functions, or allow A2M to retain its protease inhibitor function and bind to proteases. This further induces conformational changes, facilitating cellular entry for functional execution or lysosomal degradation. Notably, if A2M first binds to proteases as a protease inhibitor, its molecular chaperone function is suppressed. The broken red line represents the bait region, and the green structure represents the receptor-binding domain. Arrows indicate mechanistic processes, while T-shaped arrows represent inhibitory effects.

As previously noted, the LRP1 receptor plays a major role in clearing A2M–protease complexes. However, LRP1 is not the only cell surface receptor for A2M. In fact, more than 40 ligands have been identified for LRP1 to date, including lipoproteins, matrix proteins, intracellular proteins, lipases, and various proteases [18]. The mechanism by which LRP1 mediates the clearance of A2M–protease complexes is implicated in the development and treatment of multiple diseases. For example, transformed A2M activates the insulin signaling pathway via binding to LRP1. This enhancement of insulin response can be significantly suppressed by knocking down LRP1 expression or inhibiting its activity, a finding with potential therapeutic relevance for cardiovascular diseases associated with hypercholesterolemia [115]. A2M can also modulate LRP1 levels on the plasma membrane of retinal glial cells by activating a Ras-related protein Rab-10-dependent exocytic pathway, suggesting a new potential target for retinal disease research [116]. During inflammation, elevated hypochlorous acid levels strengthen the binding between A2M and LRP1, allowing more effective immune regulation and reduced inflammatory damage [21]. The interaction of A2M and LRP1 is also involved in glial cell differentiation, neurotoxicity, and various aspects of neurological pathology and treatment [117,118]. Moreover, A2M binding to LRP1 influences macrophage number and function in inflammation, infection, and cancer by promoting macrophage proliferation and activating pathways such as MAPK [119,120,121]. Recent studies show that amyloid beta 1-42 enhances the activity of the cation channel Transient Receptor Potential Vanilloid 1 (TRPV1) in peripheral sensory neurons via the Low-Density Lipoprotein Receptor-Related Protein 1-Src Homology-2 Domain-Containing Protein Tyrosine Phosphatase 2 (LRP1–SHP2) pathway, increasing pain perception and inflammatory responses. Conversely, A2M inhibits TRPV1 activity and reduces heat pain sensitivity through the same LRP1–SHP2 pathway, offering valuable insights into degenerative neurological diseases and potential new analgesic and anti-inflammatory therapies [122]. Through its downstream signaling cascades, the binding of LRP1 to A2M modulates immune cell function. Multiple studies have confirmed that A2M–LRP1 interaction activates pathways such as the phosphatidylinositol 3-kinase signaling pathway (PI3K/Akt) [18], the nuclear factor κB (NF-κB) signaling pathway [123], the Kelch-like ECH-associated protein 1/nuclear factor E2-related factor 2 (Keap1/Nrf2) signaling pathway [124], the Janus kinase-signal transducer and activator of transcription (JAK-STAT) signaling pathway [108], the mitogen-activated protein kinase-extracellular signal-regulated kinase (MAPK-Erk) signaling pathway [41], generally promoting cell proliferation, migration, and protein expression and secretion (Figure 5A).

Another membrane receptor for A2M is GRP78. Also known as binding immunoglobulin protein or heat shock 70 kDa protein 5, GRP78 is a member of the HSP70 chaperone family and functions as an anti-apoptotic protein [125]. A2M binds to GRP78 via its RBD [126]. While GRP78 is constitutively expressed at low levels on normal cells, various stimuli can upregulate its surface expression. In mice, multiple triggers increase GRP78 on antigen-presenting cells, potentially influencing A2M-mediated antigen presentation [127]. Under specific conditions, A2M binding to surface GRP78 regulates macrophage migration [128]. Surface GRP78 is critical for macrophage-mediated inflammatory responses; anti-GRP78 antibodies can inhibit MAPK and NF-κB signaling, effectively suppressing macrophage inflammation [129]. Additionally, GRP78 binding to transformed A2M activates downstream ERK or Akt pathways, modulating transcription factor activity and promoting cell proliferation and survival—processes that may contribute to tumor growth and metastasis [130]. Studies report that A2M–GRP78 interaction mediates prostate cancer [127,131] and hepatocellular carcinoma [132]. Transformed A2M also contributes to diabetic nephropathy pathogenesis, and blocking A2M–GRP78 binding inhibits pro-fibrotic signaling in glomerular mesangial cells, ameliorating the condition [133,134]. The A2M–GRP78 interaction also occurs in sperm, where it activates the cofilin pathway and triggers GRP78 phosphorylation, maintaining sperm structural integrity and motility and improving asthenozoospermia [128]. Beyond working through LRP1 and GRP78 in physiological regulation, signaling, and disease treatment, A2M may also help viruses and other pathogens evade immune detection. Recent work shows that A2M binding to LRP1/GRP78 facilitates Chandipura virus attachment, internalization, and evasion of A2M’s antiviral functions [135]. This finding underscores that A2M likely possesses numerous unexplored functions and mechanisms awaiting further investigation.

#### 6.2.4. The Chaperone Function of A2M

Molecular chaperones are a class of proteins that facilitate proper protein folding and prevent aberrant protein–protein interactions, playing a crucial role in maintaining intracellular protein homeostasis. Some molecular chaperones are constitutively secreted into the extracellular space [136,137], where they are termed extracellular molecular chaperones [138]. These extracellular chaperones perform surveillance functions within the extracellular matrix, identifying misfolded and aggregated proteins to facilitate the clearance of aged or damaged proteins, a critical defense mechanism against the toxicity and physical damage caused by protein aggregation [139]. French et al. first identified A2M as an ATP-independent protective molecular chaperone capable of intervening early in protein misfolding. It encapsulates misfolded proteins to form stable complexes, thereby suppressing stress-induced aggregation and precipitation [64]. When A2M initially binds a stressed or misfolded protein, this interaction does not activate A2M, and the resulting complex is not recognized by LRP1. At this stage, A2M can still interact with proteases to form a ternary stressed protein–A2M–protease complex. Subsequent protease binding induces a conformational change in A2M, enabling recognition by LRP1 (Figure 5B) [140]. This suggests that protease interaction may serve as an in vivo switch triggering LRP-mediated uptake and clearance, thereby promoting the removal of stressed protein–A2M complexes. Complexes formed between native A2M and misfolded proteins may also be internalized via other cell surface receptors (Figure 5B). However, if A2M first acts as a protease scavenger, it may lose its ability to function as a molecular chaperone (Figure 5B) [140]. As an extracellular molecular chaperone, A2M shares functional similarities with small heat shock proteins, clusterin, and ceruloplasmin [141,142]. It exhibits holdase chaperone activity by inhibiting both amorphous and fibrillar protein aggregation in vitro, maintaining protein solubility and stability without directly promoting refolding [143,144]. Hypochlorous acid has been shown to modulate A2M’s protease-binding capacity, thereby influencing its chaperone activity [21]. Recent studies indicate that A2M preferentially targets aggregation-prone misfolded proteins resulting from gradual denaturation. The resulting complexes are internalized via specific receptors and degraded lysosomally, whereas clusterin targets a broader spectrum of denatured proteins [35]. Nevertheless, the structural basis and precise molecular mechanisms underlying A2M’s chaperone function remain unclear.

Protein aggregation is widely implicated in the pathogenesis of Alzheimer’s disease and other neurodegenerative disorders [145]. Although the extracellular chaperone mechanism of A2M is not fully elucidated, evidence shows that A2M inhibits amyloid fibril formation of multiple proteins and protects cells from amyloid-β (Aβ)-induced toxicity by interacting with early prefibrillar aggregates [139]. In neuroprotection, A2M stabilizes misfolded proteins such as Aβ and aggregated α-synuclein, preventing their transformation into neurotoxic species [21]. These findings link A2M’s chaperone activity to protein deposition diseases, including neurodegenerative diseases, prion diseases, and atherosclerosis [35,146]. Endothelial dysfunction is a key factor in atherosclerosis. Changes in A2M levels correlate with flow-mediated dilation values—an important clinical indicator of atherosclerosis, suggesting that A2M may serve as a biomarker for endothelial dysfunction and atherosclerosis risk [147]. In prion diseases, A2M promotes the conversion of cellular prion protein to the protease-resistant isoform, potentially contributing to conformational changes in prion proteins through its roles in inflammation and neurodegeneration [148]. Notably, the regulatory role of A2M in protein misfolding and aggregation is not unidirectional, a complexity reflected in its involvement in neurodegenerative diseases such as Alzheimer’s disease. Transformed A2M inhibits amyloid formation through two mechanisms: first, via degradation of amyloid precursor protein by protease–A2M complexes; second, by direct chaperone action preventing further fibril formation by misfolded proteins. In the absence of proteases, activated A2M suppresses fibril formation solely through its chaperone function [22]. In early-stage Parkinson’s disease, A2M shows significantly elevated activity as a neuroprotective chaperone [149], though the underlying mechanisms require further investigation [94]. Recent studies report elevated levels of A2M, IL-1α, and IL-1β in mild cognitive impairment and Parkinson’s disease, and increased IL-1α, IL-1β, and IL-6 in both early- and late-onset Alzheimer’s patients, suggesting a positive correlation between these inflammatory markers and disease severity [150]. In Alzheimer’s brains, upregulated A2M binds LRP1, promoting Aβ endocytosis and degradation while attenuating neuroinflammation [151].

Notably, A2M exhibits dual roles, including neuroprotective and neurotoxic. It can inhibit the activity of neurotrophic factors and promote tau protein phosphorylation [152]. Blood A2M levels correlate with cerebrospinal fluid concentrations of tau and phosphorylated tau. Within the A2M gene network lies Regulator of Calcineurin 1 (RCAN1), an inhibitor of calcineurin, a tau phosphatase. RCAN1-mediated inhibition of calcineurin increases the risk of tau hyperphosphorylation, potentially exacerbating Alzheimer’s pathology [153]. Given the complex involvement of A2M in both neuroprotection and neurotoxicity, further mechanistic studies are urgently needed. At the same time, A2M represents a promising target for the prevention and treatment of neurodegenerative diseases.

#### 6.2.5. The Regulatory Role of A2M in Tumors

Proteases play a critical role in tumor growth, invasion, and metastasis, primarily by degrading the extracellular matrix and connective tissue, thereby creating a permissive microenvironment for cancer progression. Recent studies indicate that proteases are involved not only in early tumorigenesis and cancer cell growth [154], but also represent potential therapeutic targets, as modulating their activity can influence cancer development. As early as 1973, Arenne et al. observed that physiological concentrations of A2M suppressed the proliferation of L1210 leukemia cells [155]. Since then, numerous studies have explored the role of A2M in various cancers, including prostate, bladder, colon, breast, lung, and liver cancer [154,156,157]. For instance, in astrocytoma, transformed A2M (but not the native form) interacts with LRP1 and modulates cancer cell behaviors via the Wnt/β-catenin pathway, highlighting its conformation-dependent tumor-suppressive function [158]. More recently, A2M has been shown to regulate antioxidant enzymes via the Akt pathway and reduce ROS accumulation, providing a radioprotective effect in early-stage radiation-induced osteonecrosis and mitigating adverse effects in head and neck cancer patients after radiotherapy [159]. Through bioinformatic analyses, Facchiano et al. further demonstrated that elevated A2M expression correlates with improved overall survival in multiple cancers, including melanoma, suggesting its potential as a novel molecular target [160]. However, the role of A2M appears context-dependent. For example, Hsiao et al. reported that A2M overexpression enhanced proliferation, migration, and invasion in lung adenocarcinoma and impaired immune cell infiltration, thereby suppressing anti-tumor immunity [161]. Similarly, Michelis et al. found that A2M contributes to Immunoglobulin G (IgG)-hexamer formation in chronic lymphocytic leukemia, and its inhibition improved complement activity and response to immunotherapy [162]. These contrasting findings suggest that A2M may function as either a tumor suppressor or a promoter, possibly depending on cancer type and molecular context. Currently, the expression patterns and mechanisms of A2M in cancer remain incompletely understood and warrant further investigation. Beyond oncology, A2M is implicated in a range of vertebrate diseases, including cardiovascular, arthritic, and neurodegenerative conditions (Table 4), underscoring its role as a component of the innate immune system.

## 7. Conclusions

A2M, a plasma protease inhibitor belonging to the alpha-2-macroglobulin family, exhibits the ability to inhibit a broad spectrum of endopeptidases. Unlike other protease inhibitors, A2M does not destroy the active sites of proteases. It is a high-molecular-weight tetrameric glycoprotein of 720 kDa, consisting of four identical subunits, each approximately 180 kDa. This tetrameric configuration is a hallmark feature of A2M in vertebrates. The mechanism by which A2M captures and clears proteases primarily involves three functional domains: the bait region, the thioester region, and the receptor-binding region. Proteases enter the channel and bind to the bait region, triggering a conformational change from the native form of A2M to its transformed state. In this transformed state, A2M encapsulates the protease, forming a complex and exposing the receptor-binding region. This complex is specifically recognized by receptors such as LRP on hepatocytes, fibroblasts, and mononuclear phagocytes, leading to its rapid clearance via the lysosomal system. Through this mechanism, A2M clears pathological endogenous proteases as well as exogenous proteases released by invading pathogens, underscoring its role as a key component of the innate immune system in maintaining immune function and systemic homeostasis.

A2M was first identified in horseshoe crabs, which are invertebrates. Subsequent studies have revealed its presence across both invertebrates and vertebrates, where it plays a vital role in protein clearance pathways. Structural predictions and functional reviews indicate that A2M is relatively conserved between invertebrates and vertebrates and is primarily involved in innate immunity. However, the specific pathways through which A2M participates in immune responses in different invertebrates, along with its interacting proteins and associated receptors, remain poorly understood and warrant further investigation. In vertebrates, A2M not only acts as a conventional protease inhibitor, clearing both endogenous and exogenous proteases, but also interacts with cytokines and growth factors, binds membrane receptors, and serves as an extracellular molecular chaperone to help maintain cellular homeostasis.

Although the broad-spectrum protease inhibitory activity of A2M has long been recognized, several fundamental questions remain unresolved. These include the structural and mechanistic basis of its ability to bind and inhibit diverse endogenous and exogenous proteases; the molecular determinants that enable A2M to accurately recognize target proteases within complex physiological environments; the evolutionary and functional rationale behind its unique mechanism, which differs from that of conventional protease inhibitors; the physiological purpose of trapping proteases without inactivating their catalytic sites; and the structural constraints that restrict its activity specifically to endopeptidases. Furthermore, A2M’s role varies significantly across different cancers and neurological diseases, as well as in diverse body fluids and pathological contexts. Elucidating the molecular recognition patterns and specific mechanisms of A2M in disease pathogenesis will be a key focus of future research. In summary, A2M plays a crucial role in innate immunity and disease modulation, holding promising potential for future clinical applications in the treatment of related diseases.

## Figures and Tables

**Table 1:** Template Proteins and Derived Models in Homology Modeling.

Name of the Template Protein	The Templates Used	Template PDB ID	Source Organism of the Template	The Constructed Model 3
Alpha-2-macroglobulin	Alpha Fold DB model of A2MG	P01023	Human	*Homo sapiens* (XP_054227366.1)
Alpha-2-macroglobulin	Alpha Fold DB model of A2MG	P06238	Rat	*Mus musculus* (XP_011239624.1) *Rattus norvegicus* (NP_036620.2)
Alpha-2-macroglobulin-like protein 1	X-ray structure of human A2ML1	7q1y	Human	*Xenopus laevis* (XP_041426754.1)
				*Danio rerio* (XP_001923676.3)
				*Petromyzon marinus* (XP_075911300.1) *Strongylocentrotus purpuratus* (XP_030830141.1) *Scolopendra japonica* (BAR45611.1) *Octopus vulgaris* (CAI9724160.1) *Dugesia japonica* (QDF60598.1) *Schmidtea mediterranea* (SMED30004348)
Alpha-2-macroglobulin	(h-alpha2M)4 activated state	7o7p	Human	*Chelonia mydas* (EMP39670.1)
CD109 antigen	Structure of native human CD109	8s3o	Human	*Branchiostoma belcheri tsingtauense* (AEG67300.1)

**Table 2:** Binding forms of A2M with cytokines or growth factors.

Binding Forms of A2M	Related Factors	References
Native A2M	Carboxypeptidase A	[110]
Transformed A2M	TNF-α, Neurotrophin, TGF-β, Growth Hormone, IL-1β, Leptin	[54,102,105,107,111,112]
Native or Transformed A2M	BDNF, TGF-β2	[103,113]

**Table 3:** Changes in the activity of cytokines or growth factors affected by A2M.

Changes in the Activity of Factors Affected by A2M	Related Factors	References
Protective Activity	IL-6, PDGF, NGF	[98,99,100]
Inhibitory Activity	IL-1β, bFGF, TGF-β, Vascular Endothelial Growth Factor	[54,97,104,114]
No Effect on Activity	Erythropoietin	[27]

**Table 4:** Diseases associated with A2M.

Disease Type	Disease Name	References
Reproductive System Diseases	Asthenozoospermia	[128]
Musculoskeletal System Diseases	Avascular Necrosis of the Femoral Head	[105,108]
Ocular Diseases	Cataract	[104]
	Glaucoma	[163]
Inflammatory Diseases	Arthritis	[93,123,164]
	Pancreatitis	[165]
	Sepsis	[166]
	Pneumonia	[167,168]
	Acute Myocarditis	[169]
Degenerative Neurological Diseases	Parkinson’s Disease	[94,149]
	Alzheimer’s Disease	[21,22]
	Prion Diseases	[148]
	Mild Cognitive Impairment	[150]
Cardiovascular Diseases	Coronary Heart Disease	[96]
	Myocardial Infarction	[107,170]
	Atherosclerosis	[147]
	Ischemic Stroke	[171]
Diabetes	Type 1 Diabetes	[27]
	Type 2 Diabetes	[95]
	Diabetic Nephropathy	[133,134]
Hematologic Diseases	Sickle Cell Disease	[101]
	Hypercholesterolemia	[115]
	Thrombosis	[172,173]
	Hemophilia	[174]
	Sepsis	[175]
Cancer	Leukemia	[155,162]
	Astrocytoma	[158]
	Prostate Cancer	[127,131]
	Liver Cancer	[132]
	Bladder Cancer	[176]
	Colon Cancer	[154]
	Breast Cancer	[177,178]
	Lung Cancer	[154,157]
	Lung Adenocarcinoma	[161]

## Data Availability

No new data were created or analyzed in this study.

## Abbreviations

The following abbreviations are used in this manuscript:

A2M	α2-Macroglobulin
3D	Three-dimensional
Aβ	Amyloid-beta peptide
BDNF	Brain-Derived Neurotrophic Factor
bFGF	Basic Fibroblast Growth Factor
BRD	Bait Region Domain
CatG	Cathepsin G
CUB	C1r/C1s, Uegf, and Bmp1 found domain
ECAM	*Escherichia coli* α2-Macroglobulin
GRP78	Glucose Regulated Protein 78
IgG	Immunoglobulin G
IL-1β	Interleukin-1β
IL-6	Interleukin-6
LRP	Low-Density Lipoprotein Receptor-Related Protein
MCR	Macroglobulin Complement-Related
MG	Macroglobulin-like Domains
NE	Neutrophil Elastase
NGF	Nerve Growth Factor
NIE	N-terminal Induced Domain of *Escherichia coli* α2-Macroglobulin
PAMP	Pathogen-Associated Molecular Pattern
PDGF	Platelet-Derived Growth Factor
PRR	Pattern Recognition Receptor
RBD	Receptor Binding Domain
RCAN1	Regulator of Calcineurin 1
ROS	Reactive Oxygen Species
TE	Thioester
TED	Thioester Domain
TEP	Thioester-Containing Protein
TGase	Transglutaminase
TRPV1	Transient Receptor Potential Vanilloid 1
